# Effect of the Combination of *Hibiscus sabdariffa* in Combination with Other Plant Extracts in the Prevention of Metabolic Syndrome: A Systematic Review and Meta-Analysis

**DOI:** 10.3390/foods12112269

**Published:** 2023-06-05

**Authors:** Ana María García-Muñoz, Ana I. García-Guillén, Desirée Victoria-Montesinos, María Salud Abellán-Ruiz, Begoña Alburquerque-González, Fernando Cánovas

**Affiliations:** 1 Faculty of Pharmacy and Nutrition, UCAM Universidad Católica San Antonio de Murcia, 30107 Murcia, Spain; 2 Faculty of Medicine, UCAM Universidad Católica San Antonio de Murcia, 30107 Murcia, Spain; 3 Faculty of Nursing, UCAM Universidad Católica San Antonio de Murcia, 30107 Murcia, Spain; 4 Izpisua Lab, HiTech, Sport and Health Innovation Hub, UCAM Universidad Católica San Antonio de Murcia, 30107 Murcia, Spain

**Keywords:** metabolic syndrome, obesity, hypertension, dyslipidemia, polyphenols

## Abstract

Metabolic syndrome is a complex and multifactorial disorder associated with increased risk of cardiovascular disease and type 2 diabetes, exacerbated by a sedentary lifestyle and situations such as the COVID-19 pandemic. Recent studies have shown that consumption of fruits and vegetables high in polyphenols has a protective effect, reducing cardiovascular risk. *Hibiscus sabdariffa* (HS) in combination with other plant extracts has recently attracted scientists’ attention due to its potential use in the treatment of metabolic syndrome. This systematic review and meta-analysis examines the effects of HS in combination with other plant extracts on the prevention of metabolic syndrome, exploring their synergistic effects and potential as therapeutic agents. For this purpose, a systematic search of randomized clinical trials (RCTs) was conducted in four different databases and the data obtained were then used for a meta-analysis. Initially, the titles and abstracts of 1368 studies were read. From these, 16 studies were examined closely for their eligibility, and finally, seven RCTs with 332 participants were included in both the meta-analysis and the qualitative analysis. Our results show that HS in combination with other plant extracts improved anthropometric parameters, blood pressure, and lipid profile (low density lipoprotein cholesterol and total cholesterol) compared to a placebo control group. It is important to note that although this meta-analysis suggests that HS in combination with other plant extracts may have a beneficial effect on cardiovascular parameters, further research is needed to determine the optimal dose and intake duration.

## 1. Introduction

Metabolic syndrome (MetS) is a complex and multifactorial disorder characterized by a cluster of interrelated metabolic risk factors that predispose individuals to an increased risk of developing cardiovascular disease (CVD) and type 2 diabetes mellitus (T2DM) [1]. With the rising prevalence of obesity and sedentary lifestyles, MetS has become a significant global health concern, affecting up to one-third of the adult population in some regions [2,3]. The syndrome comprises a number of factors, including abdominal obesity, hyperglycemia, dyslipidemia, and hypertension [4]. Given the detrimental impact of MetS on public health and its increasing socioeconomic burden, to understand the underlying mechanisms and the develop of effective prevention strategies are of paramount importance.

MetS has a substantial impact on the quality of life of affected individuals, as it often leads to a decline in physical and mental health, reduced functional ability, and increased healthcare utilization [5]. Furthermore, the COVID-19 pandemic has contributed to a higher prevalence of MetS by exacerbating risk factors such as sedentary behavior, unhealthy eating patterns, stress, and disrupted sleep [6,7]. Consequently, the pandemic has further emphasized the urgency to address MetS and its associated health complications.

Lifestyle modifications, such as dietary changes, increased physical activity, and weight loss, have been identified as critical factors in preventing and managing MetS [8]. In addition, numerous studies have shown an association between fruit and vegetable consumption, and a reduction in mortality rates related to CVDs. The main hypothesized cause for such an improvement has been linked to the high concentration of polyphenols, such as flavonoids and carotenoids, which are present in those foods [9]. In fact, polyphenols are known antioxidants that are usually present in the human diet, found mainly in fruits, vegetables, tea, coffee, chocolate, and mushrooms. Nutraceuticals are considered as another important source of antioxidants, widely used as nutritional complements and represented by traditional medicinal herbs [10]. Despite that fact, the mechanism underlying polyphenols’ cardioprotective effect is not yet fully understood.

Focusing on polyphenols, it is worth exploring the effects of plants that are enriched with these compounds, such as *Hibiscus sabdariffa* (HS), in the context of MetS. This is why we decided to focus on HS, due to its well-documented high polyphenol content and its widespread use in traditional medicine. Studies developed by Alarcon-Aguilar et al. [11] and Lin et al. [12] have shown that the polyphenol concentration in HS is considerably higher than in other plants. Therefore, the scientific interest in HS has increased due to its potential use in the treatment of metabolic syndrome [13,14].

Besides the study of HS, our analysis explores the effects of other plant extracts that have also shown potential in managing MetS, such as *Lippia citriodora* (LC) or *Passiflora edulis*, commonly known as passion fruit (PF). The synergistic effects of HS when combined with other plant extracts have been shown to enhance its therapeutic potential in managing metabolic syndrome [15,16,17,18]. Haidari et al. [19] have investigated the combination of HS with *Carum Carvi* L., also known as caraway, and they observed that the hydro-alcoholic extracts of these two plants led to a significant decrease in fasting blood glucose levels. Interestingly, this study showed that while the combination of these two extracts had a hypoglycemic effect, the effect of HS was much more potent, even normalizing the blood glucose levels in some cases. Furthermore, Moyano et al. [20] have examined the anti-obesity potential of dietary fiber from HS and *Agave tequilana*, as an additive in the feed of animals subjected to a high-fat diet. The results of Moyano et al.’s studies revealed that supplementation with HS and *Agave tequilana* was associated with a reduction in body weight, adiposity, total plasma cholesterol, and glucose levels. Recently, numerous meta-analyses have been conducted to investigate how HS can decrease blood pressure levels [21,22,23], regulate dyslipidemia [24,25,26], and reduce other cardiovascular risk factors [21]. However, to the best of our knowledge, no meta-analyses have been conducted yet to examine the synergistic effects of HS when combined with other plant extracts.

The choice to investigate HS combined with other plant extracts is further justified by a potential for broader therapeutic benefits. The mix of polyphenols and other bioactive compounds in these plants might act together to provide a more comprehensive approach to MetS management.

Therefore, this systematic review and meta-analysis aim to investigate the combined effects of HS with other plant extracts on the prevention of MetS. Recent studies have shown the potential benefits of these botanicals individually in the context of MetS prevention [27,28,29]. By conducting a comprehensive analysis of the available literature, this review seeks to shed light on the combined efficacy of these plant extracts in mitigating MetS risk factors and to provide insight into their potential as therapeutic agents in MetS prevention.

## 2. Materials and Methods

This systematic review was carried out following the preferred reporting items for systematic review and meta-analyses (PRISMA) recommendations [30]. The randomized clinical trials (RCTs) included in this study were those in which the efficacy of the combination of HS with other plant extracts in reducing markers related to cardiovascular risk was estimated. This systematic review and meta-analysis was submitted to the international prospective register of systematic reviews (PROSPERO) (registration number: CRD42023396404).

### 2.1. Eligibility Criteria

The following criteria were established for study selection based on the PICOS framework:

P (participants): healthy individuals or patients diagnosed with concomitant illnesses, such as stage I hypertension; I (intervention): consumption of HS in combination with other plant extracts; C (comparison): control groups receiving a placebo or no treatment; O (outcomes): markers related to cardiovascular risk (i.e., anthropometric parameters, blood pressure, lipid metabolism, and blood glucose); S (study type): randomized controlled trials. Searching was restricted to articles written in the English or Spanish languages which were published in peer-reviewed journals.

The exclusion criteria were: (a) studies that compared the effects of HS combined with other plant extracts while simultaneously incorporating different drug interventions; (b) studies that lacked necessary quantitative data which were required for the meta-analysis, such as means and standard deviations of the measured outcomes; (c) non-placebo-controlled studies; (d) studies using common database sources from surveys/studies, in order to no obtain redundant results; (e) case reports, editorials, opinion pieces, and reviews; and (f) duplicated studies, which refers to multiple publications reporting on the same study or patient cohort.

### 2.2. Information Sources and Search Strategy

A systematic search was performed by two researchers across PubMed, Scopus, Web of Science, and the Cochrane Database, with a defined date range spanning from January 2010 to May 2023. The study selection was guided by a structured search strategy that utilized a combination of specific keywords and Boolean operators. These keywords were grouped into five categories and each one represents a unique concept: (a) the plant *Hibiscus sabdariffa*, (b) the plant *Lippia citriodora*, (c) the general concept of plant extracts, (d) the health conditions related to metabolic syndrome, and (e) the types of studies we were interested in. The actual search query was as follows: (“*Hibiscus sabdariffa*” OR “roselle” OR “sorrel” OR “karkade” OR “flor de Jamaica”) AND ((“*Lippia citriodora*” OR “*Aloysia citrodora*” OR “lemon verbena” OR “vervain”) OR “plant extracts” OR “herbal extracts” OR “phytotherapy” OR “herbal medicine” OR “medicinal plants”) AND (“cardiovascular risk” OR “metabolic syndrome” OR diabetes OR “type 2 diabetes” OR “type II diabetes” OR “T2D” OR hypertension OR “high blood pressure” OR “elevated blood pressure” OR dyslipidemia OR lipidemia OR “lipid profile” OR “lipid disorder” OR “cholesterol levels” OR weight OR obesity OR “body mass index” OR “BMI” OR “body weight” OR “body composition” OR “insulin resistance” OR “glucose intolerance” OR “waist circumference” OR “triglycerides” OR “HDL cholesterol” OR “LDL cholesterol”) AND (“clinical trial” OR “randomized controlled trial” OR “RCT” OR “intervention study” OR “controlled trial”).

### 2.3. Selection Process

After identifying eligible studies, Mendeley (version for Windows 10; Elsevier, Amsterdam, The Netherlands) was used to remove duplicate publications. The following selection process was carried out independently by two members of the research team, who looked over each title and abstract to find possible publications that needed to be read again in the full-text phase. A third researcher was in charge of resolving discrepancies between authors.

### 2.4. Data Items and Quality Assessment

A comprehensive extraction of relevant variables was conducted. This included cardiovascular risk factors, study type, sample characteristics, selection criteria, and details of the intervention (i.e., active compounds, administration method, and duration). The extraction was performed by one researcher (D.V.-M.), and independently checked for accuracy by a second researcher (A.M.G.-M.). In cases of discrepancy, a third researcher (A.I.G.-G.) provided a final review.

The Cochrane risk-of-bias tool (RoB 2.0) [31] was employed to evaluate the risk of bias in the included studies. The tool examines five domains: (1) bias arising from the randomization process; (2) deviations from intended interventions; (3) missing outcome data; (4) measurement of the outcome; and (5) selection of the reported result. The risk of bias was independently assessed by two researchers (D.V.-M. and A.M.G.-M.), with the selection of the appropriate RoB 2.0 tool based on the type of study (parallel or crossover). [32].

The presence of publication bias was evaluated both visually and statistically. The visual assessment was conducted using a funnel plot, which allows the identification of potential bias in the meta-analysis. In addition, a more rigorous statistical evaluation was performed using Egger’s test [33], with the significance level set at 0.10.

### 2.5. Synthesis Methods

In order to analyze the effects of HS in combination with other plant extracts on markers associated with a reduction in cardiovascular risk, we conducted a set of meta-analyses using either the DerSimonian and Laird method or the inverse of the variance, depending on the chosen methodology (fixed or random effects) [34]. These analyses compared the combination treatment of HS and other plant extracts with a control group receiving either a placebo, a substance resembling the combination treatment but lacking the active ingredients, or no treatment at all.

To graphically depict the results, forest plots were generated along with the associated 95% confidence intervals (CI). Standardized mean difference (SMD) and 95% CI were computed for each study, classifying the SMD as small (0–0.20), medium (>0.20 to 0.50), or large (>0.50). Negative values for anthropometric variables such as body mass index (BMI), body weight, and body fat mass percentage, blood pressure variables including systolic blood pressure (SBP) and diastolic blood pressure (DBP), lipid metabolism markers including low-density lipoprotein cholesterol (LDL), total cholesterol (TC), and triglycerides (TG), and blood glucose were considered as indicators of a reduction in cardiovascular risk. Conversely, positive values for the high-density lipoprotein cholesterol (HDL-c) variable indicate a similar beneficial effect.

Heterogeneity among those clinical trials included in this meta-analysis was evaluated utilizing the I^2^ statistic, which was classified as not significant (<40%), moderate (40–60%), substantial (60–75%), and considerable (75–100%) [35]. If I^2^ was not statistically significant (*p* > 0.05), the fixed effects model was used for statistical analysis. On the other hand, if I^2^ was statistically significant, the random effects model was used instead.

From studies that show the treatment outcomes divided into different groups, a combination of them was perfomed, obtaining a unique group of results. In addition, standard deviations were obtained from standard errors when necessary. Such a combination of results was performed following specifications from the Cochrane handbook [36].

Statistical analyses were conducted using Stata (version 16.1; StataCorp, College Station, TX, USA). The statistical significance level was set at *p* < 0.05.

## 3. Results

### 3.1. Study Selection

**Figure 1:**
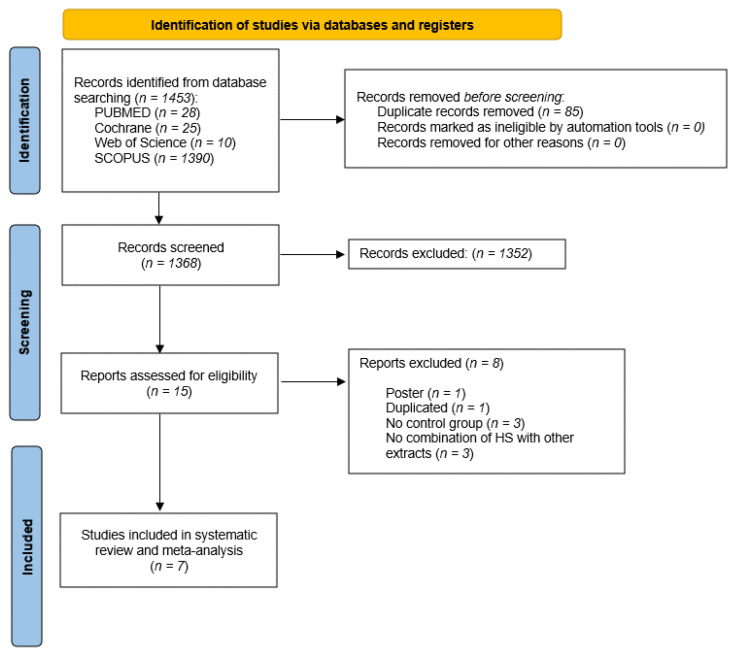
Flowchart showing study selection process.

A total of 1453 records were identified through database queries (Figure 1). After removing the duplicates, 1368 records remained, and a further screening led to the exclusion of 1352 publications. This substantial exclusion was based on the titles and abstracts, which upon careful review were determined to be unrelated to the specific objective of this review. For the evaluation, 16 studies were selected, from which 7 were excluded. Three of the identified studies were excluded because they did not combine HS extract with other plant extracts [37,38,39], and another three were excluded due to the lack of a control group [40,41,42]. One of them [43] was excluded as duplicate, and the other one [44] because it was an abstract. A total of seven studies [15,16,17,18,45,46,47] were finally included in this systematic review and meta-analysis.

### 3.2. Study Characteristic

Table 1 summarizes the main characteristics of the seven studies included in the systematic review and meta-analysis. All of them consisted of randomized clinical trials. Six of the included studies were developed following a parallel design [15,17,18,45,46,47] and the seventh study following a crossover design [16]. A total of 332 participants (59% women), with a mean age of 41.2 ± 10.3 years old (18–75 years old), were considered for this systematic review and meta-analysis. In terms of geographical regions, five of the studies were conducted in Spain [15,16,45,46,47] and the two remaining studies were carried out in Asia [17,18]. Data on BMI were reported in all of the consulted studies [15,16,17,18,45,46,47]. The information on blood pressure measurements, specifically SBP and DBP, was extracted from six different studies [15,16,17,18,45,46,47]. In relation to lipid profile details, TC and LDL-c were also available in six independent studies [16,17,18,45,46,47]. Furthermore, data on HDL-c, TG, and blood glucose were provided in five different studies [16,17,45,46,47]. Lastly, weight measurements were found in five of the included studies [15,18,45,46,47]. Data on body fat mass percentage were reported in three of the seven studies included in our research [15,45,46]. In three of the clinical trials, two capsules of HS-LC extracts, at a weight ratio (*w*/*w*) of 35:65, were administered daily [16,45,46], Marhuenda et al. [47] administered a capsule of the same weight proportion per day, and Boix-Castejón et al. [15] administered a capsule composed of a combination of 65% HS and 35% LC daily. However, in the study by Lee et al. [18], the form of administration was the consumption of tea for 8 weeks, which was composed not only of HS but also included other plant extracts such as *Perilla frutescens*, *Crataegi fructus*, *Prunus mume*, *Dolichos lablab*, and *Vigna umbellate* at a ratio of 5:6:10:2:4:4, respectively. The dosing regimen was a 3 g sachet of powder per administration, 3 times per day. On the other hand, in the study by Khongrum et al. [17], the extract was consumed through a jelly drink that contained HS and PF. The yield from the HS aqueous extraction process was 44.53% (*w*/*w*) of the dry powder, a concentration which was incorporated into the jelly drink.

### 3.3. Risk of Bias in Included Studies

**Figure 2:**
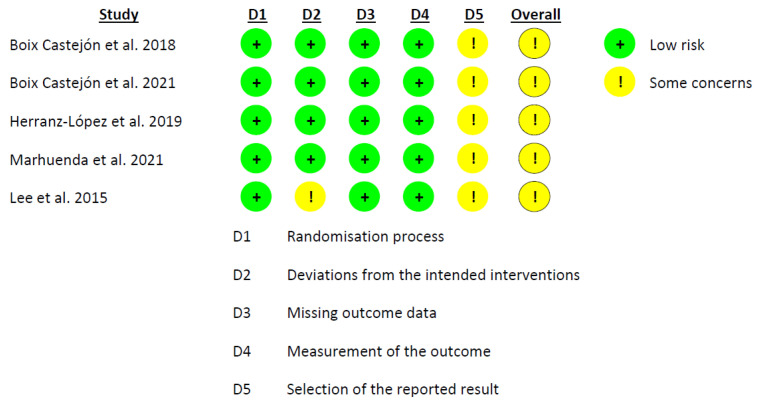
Graph of RoB 2.0, showing the analysis of the risk of bias for the parallel *per protocol* studies included in the systematic review [15,18,45,46,47].

**Figure 3:**
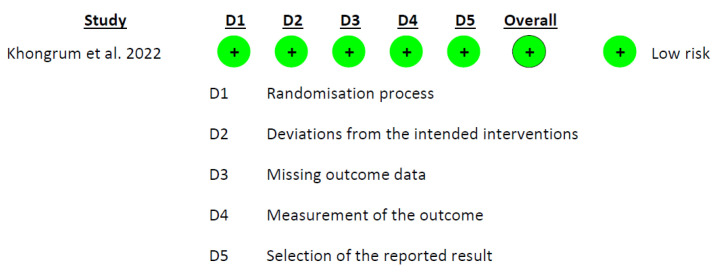
Graph of RoB 2.0, showing the analysis of the risk of bias for the parallel *intention to treat* studies included in the systematic review [17].

**Figure 4:**
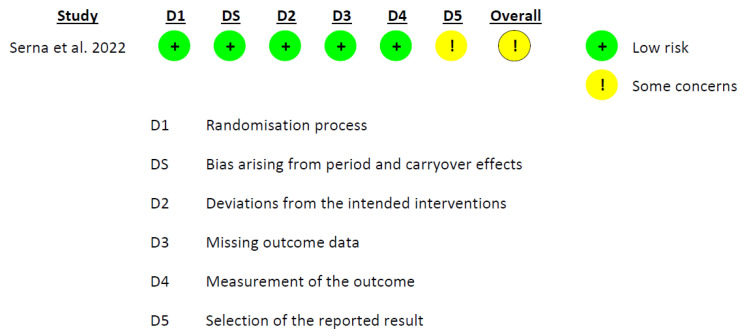
Graph of RoB 2.0, showing the analysis of the risk of bias for the crossover *per protocol* studies included in the systematic review [16].

The evaluation of all of the included studies revealed that the overall risk of bias was uncertain. A notably persistent issue arose in domain 5, ‘selection of the reported result’, where ‘some concerns’ were identified in six out of the seven studies. This prevalence is not simply high, it might suggest a substantial risk of bias in this domain. The study of Lee et al. [18] also exhibited “some concerns” in domain 2, “deviations from the intended interventions”. Analyzing the rest of the domains, a low risk of bias was observed (Figure 2, Figure 3 and Figure 4).

### 3.4. Effects of the Intervention

This review assessed the effects of HS combined with other plant extracts on various parameters associated with MetS. These parameters included BMI, body weight, body fat percentage, blood pressure, lipid profile, and blood glucose levels.

#### 3.4.1. Anthropometric Parameters

The analysis included in this review, encompassed all studies assessing the impact of HS combined with other plant extracts on BMI [15,16,17,18,45,46,47]. While a significant decrease in BMI was noted in several studies [15,17,18,46,47], no significant effect was observed in two particular ones [16,45]. The meta-analysis further substantiated these observations, showing a significant reduction in BMI (SMD −0.52; 95% CI: −0.72 to −0.31; *I*^2^ = 41.51%; *p* = 0.00).

On the other hand, five studies [15,18,45,46,47] also investigated the effect on body weight and, similar to BMI, a significant decrease was reported. The meta-analysis confirmed this decrease (SMD −0.90; 95% CI: −1.74 to −0.05; *I*^2^ = 90.30%; *p* = 0.04).

**Figure 5:**
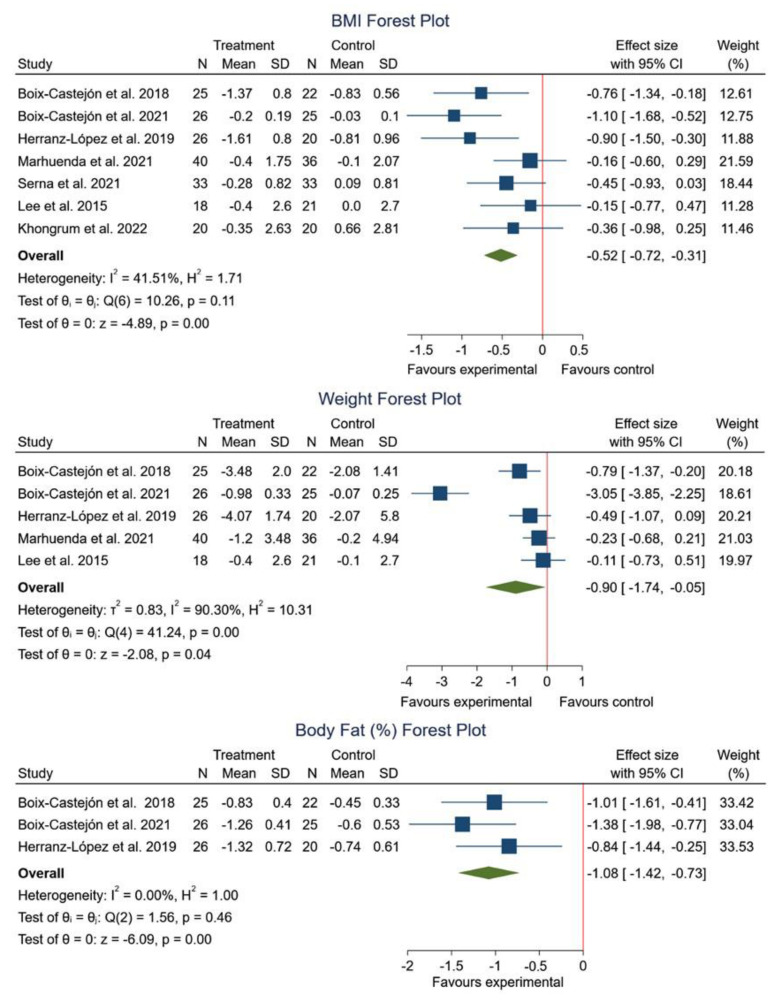
Forest plot comparisons of effects of HS in combination with other plant extracts versus a placebo on anthropometric parameters [15,16,17,18,45,46,47].

Finally, three studies assessed the impact on body fat mass percentage [15,45,46] and a significant decrease was observed. The meta-analysis validated these findings (SMD −1.08; 95% CI: −1.42 to −0.73; *I*^2^ = 0.00%; *p* = 0.00) (Figure 5).

#### 3.4.2. Blood Pressure

**Figure 6:**
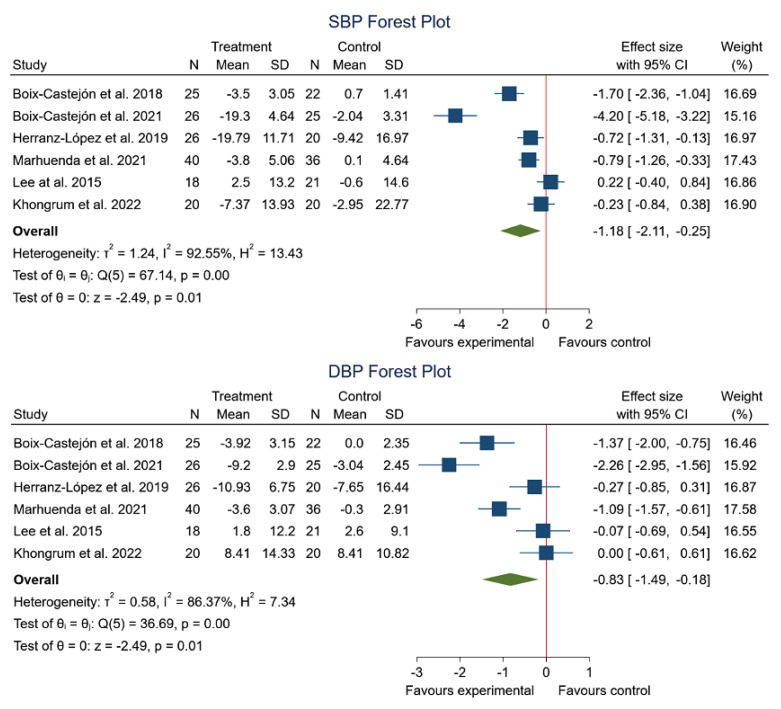
Forest plot comparisons of effects of HS in combination with other plant extracts versus a placebo on systolic and diastolic blood pressure [15,17,18,45,46,47].

The effect on blood pressure after consumption of HS in combination with other plant extracts in comparison with the placebo intake was developed in six of the seven articles included in this systematic review [15,17,18,45,46,47]. In five of the studies [15,17,45,46,47], it was observed that the participants experienced a decrease in the blood pressure measurement after consumption of the plant extract. Furthermore, this meta-analysis shows a significant decrease in SBP (SMD −1.18; 95% CI: −2.11 to −0.25; *I*^2^ = 92.55%; *p* = 0.01) and DBP (SMD −0.83; 95% CI: −1.49 to −0.18; *I*^2^ = 86.37%; *p* = 0.01). The data are represented in Figure 6.

#### 3.4.3. Lipid Profile

Six studies were evaluated for the effect of HS with other plant extracts on the lipid profile [16,17,18,45,46,47]. Four studies noted a significant decrease in LDL-c post-consumption [16,17,45,46]. Additionally, one study reported a significant decrease in TC [46], and other studies noted a significant increase in HDL-c [16].

**Figure 7:**
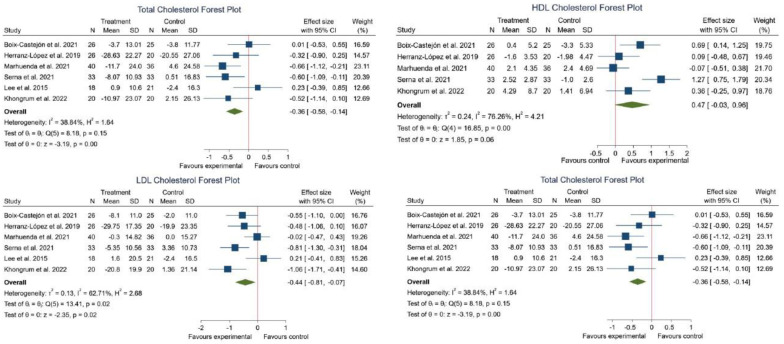
Forest plot comparisons of effects of HS in combination with other plant extracts versus a placebo on lipid profile [16,17,18,45,46,47].

The meta-analysis corroborated these results, showing a significant decrease in TC (SMD −0.36; 95% CI: −0.58 to −0.14; *I*^2^ = 38.84%; *p* = 0.00) and LDL-c (SMD −0.44; 95% CI: −0.81 to −0.07; *I*^2^ = 62.71%; *p* = 0.02). However, our results demonstrate that there was not a significant effect from the plant intake on triglyceride and HDL-c levels (Figure 7).

#### 3.4.4. Glycemia (Blood Glucose Level)

**Figure 8:**
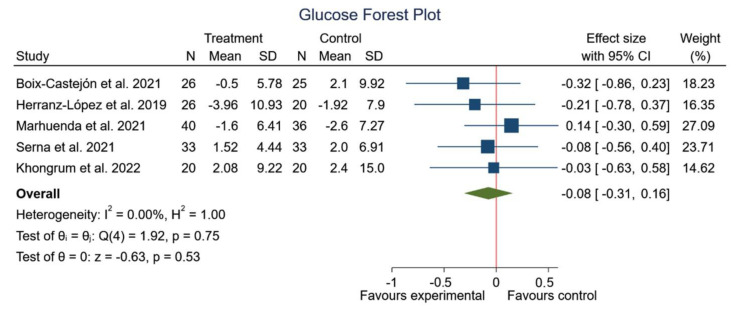
Forest plot comparisons of effects of HS in combination with other plant extract versus a placebo on glycemic profile [16,17,45,46,47].

Statistically significant results were not obtained in any of the studies consulted for the evaluation of the effects of HS in combination with other plant extracts on blood glucose [16,17,45,46,47]. Therefore, the meta-analysis shows no significant changes to the glycemic level from before to after the extract intake (SMD −0.08; 95% CI: −0.31 to 0.16; *I*^2^ = 0%; *p* = 0.53) (Figure 8).

### 3.5. Publication Bias

The funnel plot of standard error versus effect size (weighted mean difference) for BMI, body fat mass percentage, TC, and glycemic level showed no evidence of publication bias; however, body weight, SBP, DBP, HDL-c, LDL-c, and triglycerides appeared outside the funnel plot (Figures S1–S4). In addition, after the application of Egger’s test, the results indicated no potential publication bias for BMI (*p* = 0.14), body fat mass percentage (*p* = 0.21), HDL-c (*p* = 0.76), LDL-c (*p* = 0.61), DBP (*p* = 0.47), and glycemic level (*p* = 0.41), but it showed a statistically significant publication bias regarding the body weight (*p =* 0.005), SBP (*p =* 0.003), TC (*p =* 0.07), and triglycerides (*p =* 0.011).

## 4. Discussion

This systematic review and meta-analysis summarizes the effects of HS in combination with other plant extracts on cardiovascular risk parameters, which are closely related to metabolic syndrome. These parameters include anthropometric parameters, blood pressure, lipid profile, and blood glucose levels in subjects presenting as overweight or obese. The results show that HS in combination with other plant extracts significantly reduced anthropometric profiles and blood pressure (SBP and DBP), and improved the lipid profile as well (TC and LDL-c). Nevertheless, there were no significant improvements in the HDL-c, triglyceride, and blood glucose levels.

### 4.1. Effect of HS in Combination with Other Plant Extracts on Anthropometric Parameters

The comprehensive analysis conducted on the available literature during the development of this study indicates that, HS in combination with other plant extracts had a statistically significant impact on the improvement of anthropometric parameters. However, the mechanisms by which HS in combination with other plant extract polyphenols, such as PF or LC extract, could lead to weight loss and fat tissue reduction are still not well understood. Several proposed mechanisms of some polyphenols such as, e.g., quercetin, verbascoside, rosmarinic acid, etc., match with the generally accepted therapeutic approach towards MetS management.

First, they may modulate the expression of enzymes involved in lipid metabolism, such as lipases and fatty acid synthase, thus decreasing fat storage and increasing fatty acid oxidation [48,49]. This action supports the holistic approach to MetS prevention. Secondly, their anti-inflammatory properties and capacity to reduce the oxidative stress in adipose tissue, could also lead to an improvement of the insulin sensitivity and a more effective glucose metabolism [50,51]. These pathways are essential in maintaining metabolic homeostasis, a crucial aspect of MetS prevention. Ultimately, the polyphenols’ effects seem to reduce cell division in adipose tissue [52,53].

Bioactive compounds found in HS, LC, PF, and other plant extracts have also shown the capacity to reduce oxysterols in bile acid metabolism as well as the ability to inhibit lipid deposition in the liver [29,54]. Finally, these compounds also play a role in regulating fat absorption by increasing fecal excretion of palmitic acid [54], contributing, through a potential reduction in the obesity, to the overall aim of MetS prevention.

### 4.2. Effect of HS in Combination with Other Plant Extracts on BP

In this systematic review and meta-analysis, a statistically significant decrease in the systolic and diastolic blood pressure has been shown after intake of HS combined with other plant extracts. Some in vitro and in vivo studies have demonstrated the antihypertensive effect of HC in combination with other plant extracts and attempted to explain its mechanism of action. Ojeda et al. [55] suggest that the antihypertensive effect of HS may be due to the action of its polyphenols on the renin-angiotensin-2-aldosterone system. In particular, delfinidin-3-O-sambubiosides and cyanidin-3-O-sambubiosides anthocyanins would inhibit angiotensin-converting enzyme (ACE), leading to a reduction in blood pressure. In addition, Sarr et al. [56] reported that HS extract can induce endothelium-dependent vasodilation through the activation of the phosphatidylinositol 3-kinase/Akt pathway, leading to phosphorylation of endothelial nitric oxide synthase (eNOS).

Regarding other plant extracts, Lewis et al. [57] found that the administration of *Passiflora edulis* extract reduced blood pressure in spontaneously hypertensive rats, highlighting the potential efficacy of the combination of these plant extracts in mitigating MetS risk factors. Among flavonoids involved in lowering blood pressure, phenolic acids (especially protocatechuic acid) [58], flavonols (quercetin) [59], organic acid (hydroxycitric acid and hibiscus acid) [60], and anthocyanins (delphinidin-3-sambubioside and cyanidin-3-sambubioside) [56] are the most active ones.

Focusing on the action of LC extract, the phenylpropanoids verbascoside, isoverbascoside, and rosmarinic acid are among the most abundant polyphenols in this extract [61], showing a similar mechanism of action (by inhibiting ACE activity) to those included in HS extract [62,63]. LC extract could be involved in the nitric oxide (NO) pathway [64]. LC phenolic compounds increase NO production, a potent vasodilator which produces an improvement in blood flow and a decrease in blood pressure [65].

The findings presented in this study coincide with several meta-analyses that have evaluated the effect of HS intake on blood pressure showing promising results. For example, a recent meta-analysis explains that the intake of HS tea or extract can significantly reduce systolic and diastolic blood pressure [66]. In addition, a systematic review and meta-analysis of 13 clinical trials comparing HS with antihypertensive drugs, found that the extract was effective in lowering SBP and DBP in patients with mild to moderate hypertension. However, this effect was not greater than the one obtained using other kinds of antihypertensive drugs [67]. These findings further support the potential role of HS combined with other plant extracts as therapeutic agents in MetS prevention.

### 4.3. Effect of HS in Combination with Other Plant Extracts on Lipid Profile

This systematic review and meta-analysis shows significant results regarding the impact of HS in combination with other plant extracts on the lipid profile. A statistically significant decrease in the total cholesterol and LDL-c parameters was observed, which is consistent with the potential benefits of these botanicals individually in the context of MetS prevention. However, there were no significant improvements in HDL-c and triglyceride levels. As previously commented, the findings of this study agree with other studies in the literature, highlighting the combined efficacy of these plant extracts in mitigating MetS risk factors. Zhang et al. [24] showed that the consumption of HS by subjects with MetS significantly reduced serum total cholesterol levels, by an average of 14.66 mg/dL, and LDL-c levels, by an average of 9.46 mg/dL. Similarly, Yoo et al. [68] investigated the effects of hawthorn (*Crataegus pinnatifida*) extract on lipid profiles and its antioxidant properties in ovariectomized rats. The group of subjects consuming hawthorn fruit extract showed a significant decrease in total serum and LDL cholesterol levels compared to the control group.

Although the exact mechanism of action of these two extracts on the lipid profile is unknown, after the development of some in vitro and in vivo models, the inhibition of the enzyme 3-hydroxy-3-methylglutaryl-coenzyme A (HMG-CoA) reductase and the stimulation of LDL-c receptor expression are considered as possible explanations. These two effects produce a decrease in the cholesterol synthesis rate and an increase in the cholesterol clearance from the body, respectively [69,70]. These findings further support the potential of HS combined with other plant extracts as a therapeutic agent in MetS prevention, providing valuable insights into its mechanisms of action.

### 4.4. Effect of HS in Combination with Other Plant Extracts on Blood Glucose

Despite the fact that this meta-analysis does not support the idea of a decrease in blood glucose in overweight and obese individuals after the consumption of HS in combination with other plant extracts, in many other studies, the role of phenolic compounds in the regulation of glucose levels through various mechanisms of action has been observed.

To illustrate, Bule et al. [25] conducted a systematic review and meta-analysis of eight randomized clinical trials, and showed a reduction in fasting blood glucose levels and TC levels, demonstrating a positive effect of HS on blood sugar levels and lipid metabolism. Likewise, Boushehri et al. [66] showed that HS sour tea consumption is associated with a significant reduction in fasting plasma glucose. Therefore, both the anthocyanins of HS, as well as verbascoside, isoverbascoside, and rosmarinic acid of LC, seem to have hypoglycemic effects [71,72,73,74]. One of the accepted mechanisms of action of these extracts is the improvement of insulin sensitivity, which is crucial for maintaining healthy blood glucose levels. Several studies have shown that anthocyanins can enhance insulin sensitivity and glucose uptake by stimulating the activation of the insulin signaling pathway in pancreatic cells [50,51]. Another mechanism involved in this action is the inhibition of alpha-glucosidase and alpha-amylase, two enzymes that play an important role in the digestion and intestinal absorption of carbohydrates [75]. Some studies have shown that anthocyanins can inhibit these enzymes, producing a reduction in the postprandial glucose spike and an improvement of glucose metabolism [71,76]. Furthermore, the antioxidant effect of polyphenols, which can protect against oxidative stress and inflammatory effects in the complete body [77,78], is also considered as a key mechanism of action of these compounds. As is observed in our study, these processes show the therapeutic potential of the extracts in MetS prevention.

### 4.5. Limitations

This systematic review and meta-analysis contains several limitations which should be recognized. Firstly, the specific mechanisms through which HS in combination with other plant extracts induce weight loss and fat tissue reduction are not fully understood. As a result, providing a comprehensive explanation of the effects on anthropometric parameters, blood pressure, lipid profile, and blood glucose levels poses a significant challenge. Secondly, the sample size for the meta-analysis is relatively small, which may introduce bias to the obtained results. Moreover, there is a potential heterogeneity among the included studies, such as differences in populations, interventions, and outcome measures, which may limit the generalizability of the findings. Due to all of this, future research should focus on gaining a better understanding of the mechanisms underlying the effects of HS in combination with other plant extracts’ polyphenols on anthropometric parameters, blood pressure, lipid profile, and blood glucose levels in overweight and obese individuals. To reach this goal, more in-depth investigations into the molecular pathways involved, as well as examining the impact of individual polyphenols or their combinations should be performed. Additionally, larger and more homogeneous sample sizes should be employed to reduce the potential for bias and improve the reliability of the results. Lastly, comparisons with other interventions or therapies, such as antihypertensive drugs or dietary approaches, could also provide valuable insights into the effectiveness of HS in combination with other plant extracts in managing cardiovascular risk factors.

## 5. Conclusions

This systematic review and meta-analysis suggests that HS in combination with other plant extracts may have potential benefits by improving anthropometric parameters (weight loss and fat tissue reduction), blood pressure levels, and lipid profiles (total cholesterol and LDL-c) in individuals, whether they are healthy or have certain conditions such as hypertension or obesity.

Beyond the scope of these specific conditions, the potential health benefits of HS and other plant extracts may have broader implications for the general population’s health. The demonstrated improvements in metabolic health markers coincide with the global health goals of reducing non-communicable diseases, such as cardiovascular diseases and type 2 diabetes. Furthermore, the potential weight loss benefits could be an important tool in combating the global obesity epidemic. Importantly, these benefits could be particularly relevant in the current context of the COVID-19 pandemic, as both obesity and hypertension have emerged as significant risk factors for severe outcomes in patients with COVID-19. Thus, the potential of these plant extracts to ameliorate these conditions could contribute, indirectly, to improved prognoses in such individuals.

However, given the current limited scope of available studies, these findings should be interpreted as preliminary. Therefore, additional, more extensive clinical trials are needed to strengthen these evidence-based conclusions and further evaluate the role of these plant extracts in MetS prevention and management.

## Figures and Tables

**Table 1:** Characteristics of the studies included in the systematic review and meta-analysis.

Reference	Year	N	Women (%)	Age	BMI	Design RCT	Participants’ Health Condition	Active Ingredients: Content and Dose	Duration	Outcome
Boix-Castejón et al. [15]	2018	47	100	51	29.7	Double blind, Parallel	Overweight/obesewomen	LC (35%), HC (65%); 500 mg; 1 capsule per day	8 weeks	Anthropometric parameters, vital signs, appetite assessment, health-related quality of life, and peptides and hormones
Boix-Castejón et al. [45]	2021	51	NR	NR	30	Double blind, Parallel	Stage 1 hypertensive patients	LC (65%), HC (35%); 250 mg; 2 capsules per day	6 weeks	Anthropometric parameters, blood analysis, blood pressure
Herranz-López et al. [46]	2019	46	100	51.5	30.1	Double blind, Parallel	Overweight/obesewomen	LC (65%), HC (35%); 250 mg; 2 capsules per day	8 weeks	Anthropometric parameters, vital signs, appetite assessment, biochemical parameters
Marhuenda et al. [47]	2021	76	22.5	34.8	29.6	Double blind, Parallel	Overweight/obese subjects	LC (65%), HC (35%); 500 mg; 1 capsule per day	12 weeks	Anthropometric parameters, blood analysis, blood pressure
Serna et al. [16]	2022	33	48.48	33.8	28.1	Double blind, Crossover	Overweight/obese subjects	LC (65%), HC (35%); 250 mg; 2 capsules per day	8 weeks	Anthropometric parameters, blood analysis, appetite assessment
Lee et al. [18]	2015	39	51.3	49.5	11.7	Double blind, Parallel	Adults with dyslipidemia	PFR, CF, PM, DL, VU, and HS at a ratio of 6:10:2:4:4:5, respectively	8 weeks	Anthropometric parameters, blood analysis, blood pressure
Khongrum et al. [17]	2022	40	72.5	36.5	4.9	Double blind, Parallel	Adults with dyslipidemia	300 mL jelly drink (44.53% *w*/*w* HS and 12.12% *w*/*w* PF)	8 weeks	Anthropometric parameters, blood analysis, blood pressure

BMI: body mass index, CF: *Crataegi fructus,* DL: *Dolichos lablab,* HS: *Hibiscus sabdariffa*, LC: *Lippia citriodora*, NR: not reported, PF: passion fruit, PFR: *Perilla frutescens*, *PM: Prunus mume*, RCT: randomized clinical trial, VU: *Vigna umbellate.*

## Data Availability

The data used to support the findings of this study can be made available by the corresponding author upon request.

## Supplementary Materials

The following supporting information can be downloaded at: https://www.mdpi.com/article/10.3390/foods12112269/s1, Figure S1: Funnel plot for anthropometric profile; Figure S2: Funnel plot for blood pressure; Figure S3: Funnel plot for lipid profile; Figure S4: Funnel plot for blood glucose.

## References

[1] Alberti K.G.M.M., Eckel R.H., Grundy S.M., Zimmet P.Z., Cleeman J.I., Donato K.A., Fruchart J.-C., James W.P.T., Loria C.M., Smith S.C. (2009). Harmonizing the Metabolic Syndrome: A Joint Interim Statement of the International Diabetes Federation Task Force on Epidemiology and Prevention; National Heart, Lung, and Blood Institute; American Heart Association; World Heart Federation; International Atherosclerosis Society; and International Association for the Study of Obesity. Circulation.

[2] O’Neill S., O’Driscoll L. (2015). Metabolic Syndrome: A Closer Look at the Growing Epidemic and Its Associated Pathologies. Obes. Rev..

[3] Saklayen M.G. (2018). The Global Epidemic of the Metabolic Syndrome. Curr. Hypertens. Rep..

[4] Grundy S.M. (2016). Metabolic Syndrome Update. Trends Cardiovasc. Med..

[5] Ford E.S., Li C., Wheaton A.G., Chapman D.P., Perry G.S., Croft J.B. (2014). Sleep Duration and Body Mass Index and Waist Circumference among U.S. Adults. Obesity.

[6] Sidor A., Rzymski P. (2020). Dietary Choices and Habits during COVID-19 Lockdown: Experience from Poland. Nutrients.

[7] Mattioli A.V., Ballerini Puviani M., Nasi M., Farinetti A. (2020). COVID-19 Pandemic: The Effects of Quarantine on Cardiovascular Risk. Eur. J. Clin. Nutr..

[8] Grundy S.M., Cleeman J.I., Daniels S.R., Donato K.A., Eckel R.H., Franklin B.A., Gordon D.J., Krauss R.M., Savage P.J., Smith S.C. (2005). Diagnosis and Management of the Metabolic Syndrome: An American Heart Association/National Heart, Lung, and Blood Institute Scientific Statement. Circulation.

[9] Behl T., Bungau S., Kumar K., Zengin G., Khan F., Kumar A., Kaur R., Venkatachalam T., Tit D.M., Vesa C.M. (2020). Pleotropic Effects of Polyphenols in Cardiovascular System. Biomed. Pharmacother..

[10] Di Lorenzo C., Colombo F., Biella S., Stockley C., Restani P. (2021). Polyphenols and Human Health: The Role of Bioavailability. Nutrients.

[11] Alarcon-Aguilar F.J., Zamilpa A., Perez-Garcia M.D., Almanza-Perez J.C., Romero-Nuñez E., Campos-Sepulveda E.A., Vazquez-Carrillo L.I., Roman-Ramos R. (2007). Effect of *Hibiscus Sabdariffa* on Obesity in MSG Mice. J. Ethnopharmacol..

[12] Lin T.-L., Lin H.-H., Chen C.-C., Lin M.-C., Chou M.-C., Wang C.-J. (2007). *Hibiscus sabdariffa* Extract Reduces Serum Cholesterol in Men and Women. Nutr. Res..

[13] Herranz-López M., Olivares-Vicente M., Encinar J.A., Barrajón-Catalán E., Segura-Carretero A., Joven J., Micol V. (2017). Multi-Targeted Molecular Effects of *Hibiscus Sabdariffa* Polyphenols: An Opportunity for a Global Approach to Obesity. Nutrients.

[14] Pattanittum P., Ngamjarus C., Buttramee F., Somboonporn C. (2010). Roselle for Hypertension in Adults. Cochrane Database Syst. Rev..

[15] Boix-Castejón M., Herranz-López M., Pérez Gago A., Olivares-Vicente M., Caturla N., Roche E., Micol V. (2018). Hibiscus and Lemon Verbena Polyphenols Modulate Appetite-Related Biomarkers in Overweight Subjects: A Randomized Controlled Trial. Food Funct..

[16] Serna A., Marhuenda J., Arcusa R., Pérez-Piñero S., Sánchez-Macarro M., García-Muñoz A.M., Victoria-Montesinos D., Cánovas F., López-Román F.J. (2022). Effectiveness of a Polyphenolic Extract (*Lippia citriodora* and *Hibiscus sabdariffa*) on Appetite Regulation in Overweight and Obese Grade I Population: An 8-Week Randomized, Double-Blind, Cross-over, Placebo-Controlled Trial. Eur. J. Nutr..

[17] Khongrum J., Yingthongchai P., Boonyapranai K., Wongtanasarasin W., Donrung N., Sukketsiri W., Prachansuwan A., Chonpathompikunlert P. (2022). Antidyslipidemic, Antioxidant, and Anti-Inflammatory Effects of Jelly Drink Containing Polyphenol-Rich Roselle Calyces Extract and Passion Fruit Juice with Pulp in Adults with Dyslipidemia: A Randomized, Double-Blind, Placebo-Controlled Trial. Oxid. Med. Cell. Longev..

[18] Lee C.-Y., Yu M.-C., Lin C.-C., Lee M.-Y., Wei J.C.-C., Shih H.-C. (2015). Efficacy and Safety of Herbal Medicine Yun-Cai Tea in the Treatment of Hyperlipidemia: A Double-Blind Placebo-Controlled Clinical Trial. Chin. J. Integr. Med..

[19] Haidari M., Alami K., Hossaini A., Mousavi S.Y. (2020). Effect of Afghan *Hibiscus sabdariffa* L. and *Carum carvi* L. Hydro-Alcoholic Extracts Either Alone or in Combination on Blood Glucose Level in Diabetic Rats. Int. J. Ayurvedic Med..

[20] Moyano G., Sáyago-Ayerdi S.G., Largo C., Caz V., Santamaria M., Tabernero M. (2016). Potential Use of Dietary Fibre from *Hibiscus sabdariffa* and *Agave tequilana* in Obesity Management. J. Funct. Foods.

[21] Ellis L.R., Zulfiqar S., Holmes M., Marshall L., Dye L., Boesch C. (2022). A Systematic Review and Meta-Analysis of the Effects of *Hibiscus sabdariffa* on Blood Pressure and Cardiometabolic Markers. Nutr. Rev..

[22] Wahabi H.A., Alansary L.A., Al-Sabban A.H., Glasziuo P. (2010). The Effectiveness of *Hibiscus sabdariffa* in the Treatment of Hypertension: A Systematic Review. Phytomedicine.

[23] Serban C., Sahebkar A., Ursoniu S., Andrica F., Banach M. (2015). Effect of Sour Tea (*Hibiscus sabdariffa* L.) on Arterial Hypertension: A Systematic Review and Meta-Analysis of Randomized Controlled Trials. J. Hypertens..

[24] Zhang B., Yue R., Wang Y., Wang L., Chin J., Huang X., Jiang Y. (2020). Effect of *Hibiscus sabdariffa* (Roselle) Supplementation in Regulating Blood Lipids among Patients with Metabolic Syndrome and Related Disorders: A Systematic Review and Meta-Analysis. Phytother. Res..

[25] Bule M., Albelbeisi A.H., Nikfar S., Amini M., Abdollahi M. (2020). The Antidiabetic and Antilipidemic Effects of *Hibiscus sabdariffa*: A Systematic Review and Meta-Analysis of Randomized Clinical Trials. Food Res. Int..

[26] Aziz Z., Wong S.Y., Chong N.J. (2013). Effects of *Hibiscus sabdariffa* L. on Serum Lipids: A Systematic Review and Meta-Analysis. J. Ethnopharmacol..

[27] Hopkins A.L., Lamm M.G., Funk J.L., Ritenbaugh C. (2013). *Hibiscus sabdariffa* L. in the Treatment of Hypertension and Hyperlipidemia: A Comprehensive Review of Animal and Human Studies. Fitoterapia.

[28] Borrás-Linares I., Pérez-Sánchez A., Lozano-Sánchez J., Barrajón-Catalán E., Arráez-Román D., Cifuentes A., Micol V., Carretero A.S. (2015). A Bioguided Identification of the Active Compounds That Contribute to the Antiproliferative/Cytotoxic Effects of Rosemary Extract on Colon Cancer Cells. Food Chem. Toxicol..

[29] De Faveri A., De Faveri R., Broering M.F., Bousfield I.T., Goss M.J., Muller S.P., Pereira R.O., de Oliveira E Silva A.M., Machado I.D., Quintão N.L.M. (2020). Effects of Passion Fruit Peel Flour (*Passiflora edulis* f. *Flavicarpa* O. Deg.) in Cafeteria Diet-Induced Metabolic Disorders. J. Ethnopharmacol..

[30] Page M.J., McKenzie J., Bossuyt P. (2021). The PRISMA 2020 Statement: An Updated Guideline for Reporting Systematic Reviews. BMJ.

[31] Alarcón M., Ojeda R., Huaricancha I., Hilario K. Análisis Crítico de Ensayos Clínicos Aleatorizados: Riesgo de Sesgo. http://www.scielo.org.pe/scielo.php?script=sci_arttext&pid=S1019-43552015000400008.

[32] Sterne J.A.C., Savović J., Page M.J., Elbers R.G., Blencowe N.S., Boutron I., Cates C.J., Cheng H.-Y., Corbett M.S., Eldridge S.M. (2019). RoB 2: A Revised Tool for Assessing Risk of Bias in Randomised Trials. BMJ.

[33] Egger M., Smith G.D., Schneider M., Minder C. (1997). Bias in Meta-Analysis Detected by a Simple, Graphical Test. BMJ.

[34] DerSimonian R., Laird N. (1986). Meta-Analysis in Clinical Trials. Control. Clin. Trials.

[35] Borenstein M., Hedges L.V., Higgins J.P.T., Rothstein H.R. (2021). Introduction to Meta-Analysis.

[36] Higgins J.P.T., Thomas J., Chandler J., Cumpston M., Li T., Page M., Welch V. (2019). Cochrane Handbook for Systematic Reviews of Interventions.

[37] Bourqui A., Niang E.A.B., Graz B., Diop E.A., Dahaba M., Thiaw I., Soumare K., Valmaggia P., Nogueira R.C., Cavin A.-L. (2021). Hypertension Treatment with Combretum Micranthum or *Hibiscus sabdariffa*, as Decoction or Tablet: A Randomized Clinical Trial. J. Hum. Hypertens..

[38] Seck S.M., Doupa D., Dia D.G., Diop E.A., Ardiet D.-L., Nogueira R.C., Graz B., Diouf B. (2017). Clinical Efficacy of African Traditional Medicines in Hypertension: A Randomized Controlled Trial with Combretum Micranthum and *Hibiscus sabdariffa*. J. Hum. Hypertens..

[39] Jeenduang N., Sangkaew B., Chantaracha P., Chanchareonsri S., Plyduang T., Thitdee W., Samae C., Pitumanon W. (2017). APOE and CETP TaqIB Polymorphisms Influence Metabolic Responses to *Hibiscus sabdariffa* L. and Gynostemma Pentaphyllum Makino Tea Consumption in Hypercholesterolemic Subjects. Asia Pac. J. Clin. Nutr..

[40] Elkafrawy N., Younes K., Naguib A., Badr H., Kamal Zewain S., Kamel M., Raoof G.F.A., M El-Desoky A., Mohamed S. (2020). Antihypertensive Efficacy and Safety of a Standardized Herbal Medicinal Product of *Hibiscus sabdariffa* and *Olea europaea* Extracts (NW Roselle): A Phase-II, Randomized, Double-Blind, Captopril-Controlled Clinical Trial. Phytother. Res..

[41] Jaisamut P., Tohlang C., Wanna S., Thanakun A., Srisuwan T., Limsuwan S., Rattanachai W., Suwannachot J., Chusri S. (2022). Clinical Evaluation of a Novel Tablet Formulation of Traditional Thai Polyherbal Medicine Named Nawametho in Comparison with Its Decoction in the Treatment of Hyperlipidemia. Evid. Based Complement. Altern. Med..

[42] Herrera-Arellano A., Miranda-Sánchez J., Avila-Castro P., Herrera-Alvarez S., Jiménez-Ferrer J.E., Zamilpa A., Román-Ramos R., Ponce-Monter H., Tortoriello J. (2007). Clinical Effects Produced by a Standardized Herbal Medicinal Product of *Hibiscus sabdariffa* on Patients with Hypertension. A Randomized, Double-Blind, Lisinopril-Controlled Clinical Trial. Planta Med..

[43] Marhuenda J., Perez-Piñero S., Victoria-Montesinos D., Abellán-Ruiz M.S., Caturla N., Jones J., López-Román J. (2020). Correction: Marhuenda, J.; et al. A Randomized, Double-Blind, Placebo Controlled Trial to Determine the Effective-ness a Polyphenolic Extract (Hibiscus Sabdariffa and Lippia Citriodora) in the Reduction of Body Fat Mass in Healthy Subjects. Foods.

[44] Boix-Castejon M., Herranz-Lopez M., Caturla N., Roche E., Barrajon-Catalan E., Micol V. (2017). Hibiscus and Lemon Verbena Polyphenols: Assessment for Weight Management in Overweight Volunteers. Appetite Control and Satiety. Free. Radic. Biol. Med..

[45] Boix-Castejón M., Herranz-López M., Olivares-Vicente M., Campoy P., Caturla N., Jones J., Zazo J.M., Roche E., Micol V. (2021). Effect of Metabolaid^®^ on Pre- and Stage 1 Hypertensive Patients: A Randomized Controlled Trial. J. Funct. Foods.

[46] Herranz-López M., Olivares-Vicente M., Boix-Castejón M., Caturla N., Roche E., Micol V. (2019). Differential Effects of a Combination of *Hibiscus sabdariffa* and *Lippia citriodora* Polyphenols in Overweight/Obese Subjects: A Randomized Controlled Trial. Sci. Rep..

[47] Marhuenda J., Pérez-Piñero S., Arcusa R., Victoria-Montesinos D., Cánovas F., Sánchez-Macarro M., García-Muñoz A.M., Querol-Calderón M., López-Román F.J. (2021). A Randomized, Double-Blind, Placebo-Controlled Trial to Determine the Effectiveness of a Polyphenolic Extract (*Hibiscus sabdariffa* and *Lippia citriodora*) for Reducing Blood Pressure in Prehypertensive and Type 1 Hypertensive Subjects. Molecules.

[48] Zhou J.-F., Wang W.-J., Yin Z.-P., Zheng G.-D., Chen J.-G., Li J.-E., Chen L.-L., Zhang Q.-F. (2021). Quercetin Is a Promising Pancreatic Lipase Inhibitor in Reducing Fat Absorption in Vivo. Food Biosci..

[49] Wu X., He W., Zhang H., Li Y., Liu Z., He Z. (2014). Acteoside: A Lipase Inhibitor from the Chinese Tea Ligustrum Purpurascens Kudingcha. Food Chem..

[50] Belwal T., Nabavi S.F., Nabavi S.M., Habtemariam S. (2017). Dietary Anthocyanins and Insulin Resistance: When Food Becomes a Medicine. Nutrients.

[51] El-Marasy S., el Shenawy M., Moharram F., El-Sherbeeny N. (2020). Antidiabetic and Antioxidant Effects of Acteoside from Jacaranda Mimosifolia Family Biognoniaceae in Streptozotocin–Nicotinamide Induced Diabetes in Rats. Open. Access. Maced. J. Med. Sci..

[52] Kim S.G., Kim J.-R., Choi H.C. (2018). Quercetin-Induced AMP-Activated Protein Kinase Activation Attenuates Vasoconstriction Through LKB1-AMPK Signaling Pathway. J. Med. Food.

[53] Olivares-Vicente M., Sánchez-Marzo N., Encinar J.A., de La Luz Cádiz-Gurrea M., Lozano-Sánchez J., Segura-Carretero A., Arraez-Roman D., Riva C., Barrajón-Catalán E., Herranz-López M. (2019). The Potential Synergistic Modulation of AMPK by *Lippia citriodora* Compounds as a Target in Metabolic Disorders. Nutrients.

[54] Ojulari O.V., Lee S.G., Nam J.-O. (2019). Beneficial Effects of Natural Bioactive Compounds from *Hibiscus sabdariffa* L. on Obesity. Molecules.

[55] Ojeda D., Jiménez-Ferrer E., Zamilpa A., Herrera-Arellano A., Tortoriello J., Alvarez L. (2010). Inhibition of Angiotensin Convertin Enzyme (ACE) Activity by the Anthocyanins Delphinidin- and Cyanidin-3-O-Sambubiosides from *Hibiscus sabdariffa*. J. Ethnopharmacol..

[56] Sarr M., Ngom S., Kane M.O., Wele A., Diop D., Sarr B., Gueye L., Andriantsitohaina R., Diallo A.S. (2009). In Vitro Vasorelaxation Mechanisms of Bioactive Compounds Extracted from *Hibiscus sabdariffa* on Rat Thoracic Aorta. Nutr. Metab..

[57] Lewis B.J., Herrlinger K.A., Craig T.A., Mehring-Franklin C.E., Defreitas Z., Hinojosa-Laborde C. (2013). Antihypertensive Effect of Passion Fruit Peel Extract and Its Major Bioactive Components Following Acute Supplementation in Spontaneously Hypertensive Rats. J. Nutr. Biochem..

[58] Zhang S., Gai Z., Gui T., Chen J., Chen Q., Li Y. (2021). Antioxidant Effects of Protocatechuic Acid and Protocatechuic Aldehyde: Old Wine in a New Bottle. Evid. Based Complement. Altern. Med..

[59] Edwards R.L., Lyon T., Litwin S.E., Rabovsky A., Symons J.D., Jalili T. (2007). Quercetin Reduces Blood Pressure in Hypertensive Subjects. J. Nutr..

[60] Da-Costa-Rocha I., Bonnlaender B., Sievers H., Pischel I., Heinrich M. (2014). *Hibiscus sabdariffa* L.—A Phytochemical and Pharmacological Review. Food Chem..

[61] Quirantes-Piné R., Herranz-López M., Funes L., Borrás-Linares I., Micol V., Segura-Carretero A., Fernández-Gutiérrez A. (2013). Phenylpropanoids and Their Metabolites Are the Major Compounds Responsible for Blood-Cell Protection against Oxidative Stress after Administration of *Lippia citriodora* in Rats. Phytomedicine.

[62] Pierre Luhata L., Usuki T. (2022). Free Radical Scavenging Activities of Verbascoside and Isoverbascoside from the Leaves of *Odontonema strictum* (*Acanthaceae*). Bioorg. Med. Chem. Lett..

[63] Prasannarong M., Saengsirisuwan V., Surapongchai J., Buniam J., Chukijrungroat N., Rattanavichit Y. (2019). Rosmarinic Acid Improves Hypertension and Skeletal Muscle Glucose Transport in Angiotensin II-Treated Rats. BMC Complement. Altern. Med..

[64] Zhou H., Fu B., Xu B., Mi X., Li G., Ma C., Xie J., Li J., Wang Z. (2017). Rosmarinic Acid Alleviates the Endothelial Dysfunction Induced by Hydrogen Peroxide in Rat Aortic Rings via Activation of AMPK. Oxid. Med. Cell. Longev..

[65] Napoli C., Ignarro L.J. (2009). Nitric Oxide and Pathogenic Mechanisms Involved in the Development of Vascular Diseases. Arch. Pharm. Res..

[66] Najafpour Boushehri S., Karimbeiki R., Ghasempour S., Ghalishourani S.-S., Pourmasoumi M., Hadi A., Mbabazi M., Pour Z.K., Assarroudi M., Mahmoodi M. (2020). The Efficacy of Sour Tea (*Hibiscus sabdariffa* L.) on Selected Cardiovascular Disease Risk Factors: A Systematic Review and Meta-Analysis of Randomized Clinical Trials. Phytother. Res..

[67] Abdelmonem M., Ebada M.A., Diab S., Ahmed M.M., Zaazouee M.S., Essa T.M., ElBaz Z.S., Ghaith H.S., Abdella W.S., Ebada M. (2022). Efficacy of *Hibiscus sabdariffa* on Reducing Blood Pressure in Patients With Mild-to-Moderate Hypertension: A Systematic Review and Meta-Analysis of Published Randomized Controlled Trials. J. Cardiovasc. Pharmacol..

[68] Yoo J.-H., Liu Y., Kim H.-S. (2016). Hawthorn Fruit Extract Elevates Expression of Nrf2/HO-1 and Improves Lipid Profiles in Ovariectomized Rats. Nutrients.

[69] Angiolillo A., Leccese D., Palazzo M., Vizzarri F., Casamassima D., Corino C., Di Costanzo A. (2021). Effects of *Lippia citriodora* Leaf Extract on Lipid and Oxidative Blood Profile of Volunteers with Hypercholesterolemia: A Preliminary Study. Antioxidants.

[70] Showande S.J., Adegbolagun O.M., Igbinoba S.I., Fakeye T.O. (2017). In Vivo Pharmacodynamic and Pharmacokinetic Interactions of *Hibiscus sabdariffa* Calyces Extracts with Simvastatin. J. Clin. Pharm. Ther..

[71] Zulfiqar S., Marshall L.J., Boesch C. (2022). *Hibiscus sabdariffa* Inhibits α-Glucosidase Activity in Vitro and Lowers Postprandial Blood Glucose Response in Humans. Hum. Nutr. Metab..

[72] Alegbe E.O., Teralı K., Olofinsan K.A., Surgun S., Ogbaga C.C., Ajiboye T.O. (2019). Antidiabetic Activity-Guided Isolation of Gallic and Protocatechuic Acids from *Hibiscus sabdariffa* Calyxes. J. Food Biochem..

[73] Zhu F., Asada T., Sato A., Koi Y., Nishiwaki H., Tamura H. (2014). Rosmarinic Acid Extract for Antioxidant, Antiallergic, and α-Glucosidase Inhibitory Activities, Isolated by Supramolecular Technique and Solvent Extraction from Perilla Leaves. J. Agric. Food Chem..

[74] Ritter Ruas N., Carvalho Pereira A., Lopes Silva Pereira L., Mesquita Germano C., Fontes Ferreira da Cunha E., Alves de Carvalho A., Alves Lameira O., Eduardo Brasil Pereira Pinto J., Kelly Vilela Bertolucci S. (2023). Inhibition of α-Glycosidase by *Lippia dulcis* Trevir. (Verbenaceae) Preparations, Quantification of Verbascoside, and Study of Its Molecular Docking. Chem. Biodivers..

[75] Gong L., Feng D., Wang T., Ren Y., Liu Y., Wang J. (2020). Inhibitors of A-amylase and A-glucosidase: Potential Linkage for Whole Cereal Foods on Prevention of Hyperglycemia. Food Sci. Nutr..

[76] Moein S., Moein M., Javid H. (2022). Inhibition of α-Amylase and α-Glucosidase of Anthocyanin Isolated from Berberis Integerrima Bunge Fruits: A Model of Antidiabetic Compounds. Evid. Based Complement. Altern. Med..

[77] Oliveira H., Fernandes A., Brás N.F., Mateus N., de Freitas V., Fernandes I. (2020). Anthocyanins as Antidiabetic Agents—In Vitro and In Silico Approaches of Preventive and Therapeutic Effects. Molecules.

[78] Govindaraj J., Sorimuthu Pillai S. (2015). Rosmarinic Acid Modulates the Antioxidant Status and Protects Pancreatic Tissues from Glucolipotoxicity Mediated Oxidative Stress in High-Fat Diet: Streptozotocin-Induced Diabetic Rats. Mol. Cell. Biochem..

