# Supplementary figures and images for: Effect of the Combination of Hibiscus sabdariffa in Combination with Other Plant Extracts in the Prevention of Metabolic Syndrome: A Systematic Review and Meta-Analysis
Source: Foods. 2023 Jun 5;12(11):2269. doi: 10.3390/foods12112269 (PMC10253170; doi:10.3390/foods12112269)

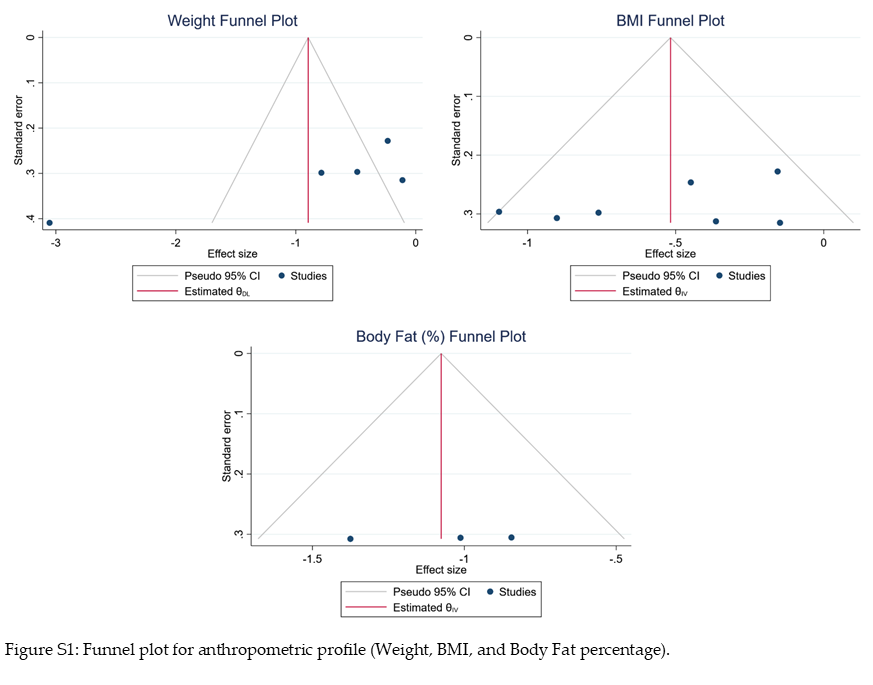

Supplement: Supplementary file 1 [file foods-12-02269-s001.zip › Figure S1.png]

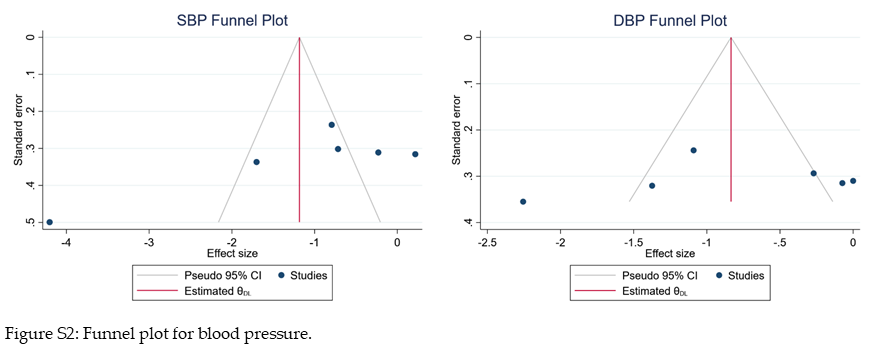

Supplement: Supplementary file 1 [file foods-12-02269-s001.zip › Figure S2.png]

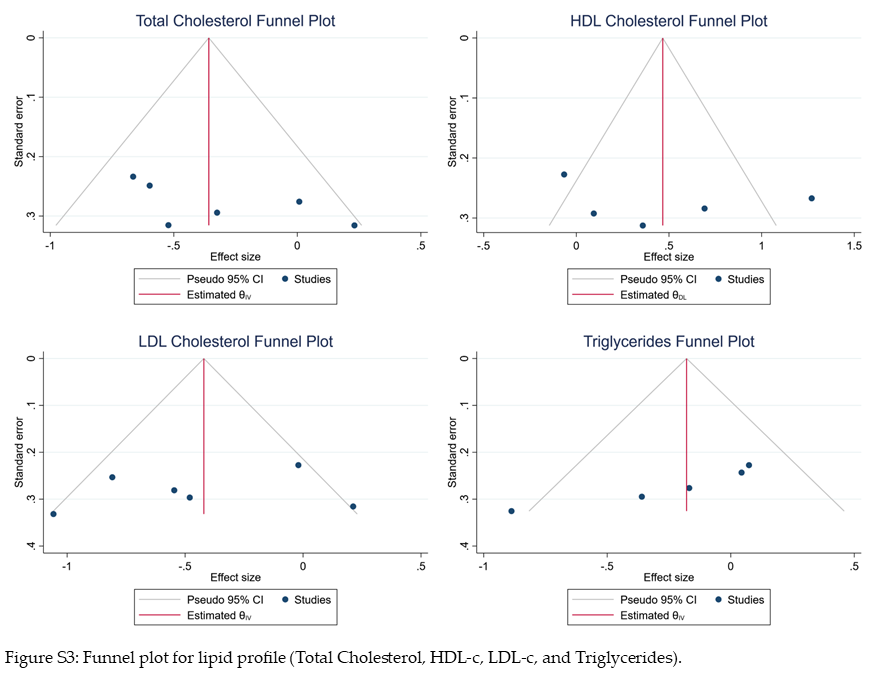

Supplement: Supplementary file 1 [file foods-12-02269-s001.zip › Figure S3.png]

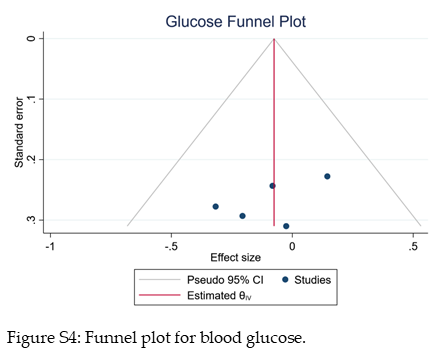

Supplement: Supplementary file 1 [file foods-12-02269-s001.zip › Figure S4.png]
